# Does the Charlson comorbidity index help predict the risk of death in COVID-19 patients?

**DOI:** 10.14744/nci.2022.33349

**Published:** 2022-04-12

**Authors:** Senol Comoglu, Aydin Kant

**Affiliations:** 1 Department of Infectious Diseases and Clinical Microbiology, SBU Umraniye Training and Research Hospital, Istanbul, Turkey; 2 Department of Chest Diseases, Trabzon Vakfıkebir State Hospital, Trabzon, Turkey

**Keywords:** Charlson comorbidity index, comorbidity, COVID-19, mortality

## Abstract

**Objective:**

Comorbidities are diseases that coexist with a disease of interest or an index disease, which can directly affect the prognosis of the disease of interest or indirectly affect the choice of treatment. The Charlson comorbidity index (CCI) is the most widely used comorbidity index. In this study, it was aimed to examine the predictive role of the CCI score on the mortality of patients with COVID-19.

**Methods:**

We have retrospectively analyzed COVID-19 patients whose diagnosis was confirmed by PCR and who were hospitalized in two centers between April 2020 and December 2020. The severity of comorbidity of the patients was categorized into five groups according to the CCI score: CCI score 0, CCI score 1–2, CCI score 3–4, CCI score 5–6, and CCI score ≥7. Factors affecting mortality and differences between groups classified by CCI were determined by logistic regression analysis and one-way analysis of variance.

**Results:**

A total of 1,559 COVID-19 patients were included in the study and 70 (4.49%) patients had deceased. Half of the study population (n=793, 50.9%) had different comorbidities. The CCI score was 3.8±2.7 in deceased patients and 1.3±1.9 in surviving individuals. There was a positive correlation between CCI scores and mortality in COVID-19 patients, with each point increase in the CCI score increasing the risk of death by 2.5%. CCI score of 4 and above predicted mortality with 87.2% sensitivity and 97.9% negative predictive value. Five (0.6%) of 766 patients with CCI scores of 0, 16 (3.6%) of 439 patients with CCI scores of 1–2, 13 (6.9%) of 189 patients with CCI scores of 3–4, and a CCI score of 5, 13 (15.7%) of 83 patients with -6 and 23 (28.0%) of 82 patients with a CCI score of ≥7 died.

**Conclusion:**

CCI is a simple, easily applicable, and valid method for classifying comorbidities and estimating COVID-19 mortality. The close relationship between the CCI score and mortality reveals the reality of how important vaccination is, especially in this group of patients. Increasing awareness of potential comorbidities in COVID-19 patients can provide insight into the disease and to improve outcomes by identifying and treating patients earlier and more effectively.

**C**OVID-19 was declared as a pandemic by the World Health Organization shortly after its emergence. The infection has resulted in the death of millions of people as of December 2021. Despite the period of more than 2 years and all the technological possibilities at our disposal, its spread and lethality still continues. Newly emerged mutant strains also contribute to this spread and mortality [1].

Advanced age and having comorbid factors cause more mortality in COVID-19, as in other infections [2]. Comorbidities are diseases that coexist with a disease of interest or an index disease, which can directly affect the prognosis of the disease of interest or indirectly affect the choice of treatment [2].

Charlson comorbidity index (CCI) is one of the most commonly used methods to evaluate comorbid factors and predict mortality. In the calculation of CCI, which has been used since 1987, many underlying conditions such as age, kidney diseases, malignancies, cerebrovascular diseases, liver diseases, and human immunodeficiency virus positivity are taken into account [3]. It can be calculated quickly and easily at the bedside since no laboratory parameters are needed. In our study, it was aimed to examine the predictive role of the CCI score on the mortality of COVID-19 patients.

## Materials and Methods

We have retrospectively analyzed COVID-19 patients whose diagnosis was confirmed by polymerase chain reaction (PCR) and who were hospitalized in two centers between April 2020 and December 2020. Demographic data of patients (gender and age), symptoms (fever, nasal congestion, cough, sputum production, shortness of breath, headache, muscle and joint pain, general weakness, nausea and vomiting, diarrhea, and smell-taste disorder), respiratory rate per minute number, results of laboratory tests at admission (hemogram and blood biochemistry), hepatorenal function, coagulation function, D-dimer, C-reactive protein (CRP), procalcitonin, ferritin, arterial blood gas analysis, chest X-ray or computerized tomography (CT) imaging findings and CO-RADS score, comorbidities, and clinical outcomes (duration of hospital stay, admission to the intensive care unit, discharge or death) were recorded in the study form. The study has been approved by The Health Sciences University, Umraniye Research and Training Hospital Clinical Research Ethics Committee at 20.12.2021 protocol number 54132726 – 000 – 27327.

Comorbidity categories were evaluated, including diabetes mellitus (DM), hypertension, heart failure, chronic heart disease, asthma, chronic obstructive pulmonary disease, chronic kidney disease, malignancy, chronic liver disease, connective tissue disease, and dementia, categorized into groups: CCI score 0, CCI score 1–2, CCI score 3–4, CCI score 5–6, and CCI score ≥7.

**Highlight key points**

• Advanced age and having comorbid factors cause more mortality in COVID-19, as in other infections.
• CCI, which has the advantage of being able to be calculated quickly at the bedside without the need for any laboratory parameters, is one of the most frequently used methods for the evaluation of comorbid factors and predicting mortality.
• CCI is a simple, easily applicable, and valid method for classifying comorbidities and estimating COVID-19 mortality.
• Each point increase in the CCI score increases the risk of death by 2.5%, and a CCI score of 4 and above can predict mortality with 87.2% sensitivity and 97.9% negative predictive value.
• The close relationship between CCI score and mortality reveals how important vaccination is in patients with comorbidities.

### Statistical Analysis

Descriptive statistical analysis was performed for all variables investigated in the study. The data were analyzed by the Statistical Package for the Social Sciences (IBM SPSS 26, New Orchard Road Armonk, Newyork 10504-12722 United States). Concordance with the normal distribution of the data obtained by the measurement was made using the Kolmogorov–Smirnov test. Student’s t-test was used for data with normal distribution, and Mann–Whitney U-test was used for data without normal distribution. Chi-square test was used in the analysis of categorical variables. Data obtained by measurement were expressed as mean±standard deviation.

The data obtained by counting were expressed as numbers (%). Pearson’s correlation test or Spearman’s correlation test was used for correlation analysis. Factors affecting mortality and differences between groups classified by CCI were determined by logistic regression analysis and one-way analysis of variance. The predictive value of CCI was determined by Receiver Operating Characteristic (ROC) analysis. P<0.05 was considered statistically significant.

## Results

A total of 1559 COVID-19 patients positive for the real-time PCR were included in the study. The mean age was 47.1±17.5 years and 53.6% were male. Symptoms were present in 87.4% of the patients. The main symptoms were fever (48.6%), cough (61.5%), shortness of breath (30.3%), diarrhea (5.5%), sore throat (10.1%), smell-taste disorder (8.9%), myalgia (13.9%), fatigue (19.6%), and headache (7.6%) (Table 1).

**Table 1.** Baseline demographics and symptoms of the study population

	% (n=1559)
Demographics	
Male	53.6
Female	46.4
Symptoms	
Yes	87.4
No	12.6
Cough	61.5
Fever	48.6
Shortness of breath	30.3
Fatigue	19.6
Myalgia	13.9
Sore throat	10.1
Smell-taste disorder	8.9
Headache	7.6
Diarrhea	5.5

Laboratory values of the patients were analyzed as follows: White blood cell (WBC) (6936±2485/μl), lymphocyte (1261±681/μl), platelet (183.4±58.9/μl), alanine aminotransferase (ALT) (53.4±76.2 U/L), aspartate aminotransferase (76.1±138.4 U/L), CRP (36.4±34.3 mg/L), lactate dehydrogenase (LDH) (481.5±132.6 U/L), D-Dimer (1026.5±1284 ng/mL), ferritin (481.6±478.9 ng/mL), and troponin (0.061±0.38μg/L).

**Figure 1.**
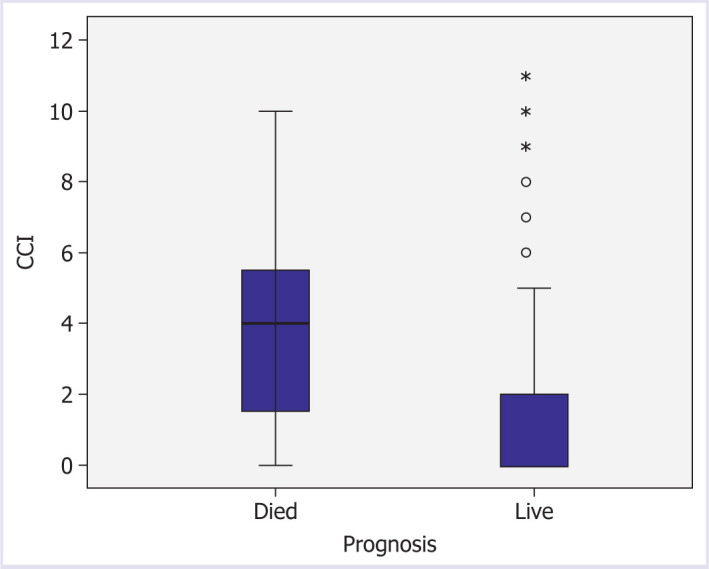
Boxplot plot of the relationship between Charlson comorbidity index score and prognosis in COVID-19 patients.

During the study period, 70 (4.49%) patients had deceased. Half of the study population (n=793, 50.9%) had different comorbidities. The CCI score was 3.8±2.7 in deceased patients and 1.3±1.9 in surviving patients (p<0.001). As CCI scores increased, mortality increased (r=0.249; p<0.001). For each point increase in CCI score, the risk of death increased by 2.5% (B=0.025 95% confidence interval [CI]: 0.20–0.30); (t=10,382; p<0.001). Five (0.6%) of 766 patients with CCI scores of 0, 16 (3.6%) of 439 patients with CCI scores of 1–2, 13 (6.9%) of 189 patients with CCI scores of 3–4, and a CCI score of 5, 13 (15.7%) of 83 patients with −6 and 23 (28.0%) of 82 patients with a CCI score of ≥7 died (p<0.001) (Table 2 and Fig. 1).

**Table 2.** Mortality rates by CCI score

CCI	n	N (mortality)	% (mortality)
0	766	5	0.6
1–2	439	16	3.6
3–4	189	13	6.9
5–6	83	13	15.7
7 or above	82	23	28

CCI: Charlson comorbidity index.

There was a positive correlation with CCI score and presence of symptoms (r=0.110; p<0.001), duration of symptoms (r=0.070; p=0.023), shortness of breath (r=0.206; p<0.001), WBC (r=0.097; p<0.001), CRP (r=0.250; p<0.001), LDH (r=0.157; p<0.001), D-dimer (0.381; p<0.001), ferritin (r=0.120; p<=0.008), and troponin (r=0.492; p<0.001). There was a negative correlation between with CCI score and smell-taste disorder (r=−0.090; p<0.001), sore throat (r=−0.118; p<0.001), headache (r=−0.066; p=0.01), lymphocyte count (r=−0.140; p<0.001), and ALT (r=−0.195; p<0.001).

**Figure 2.**
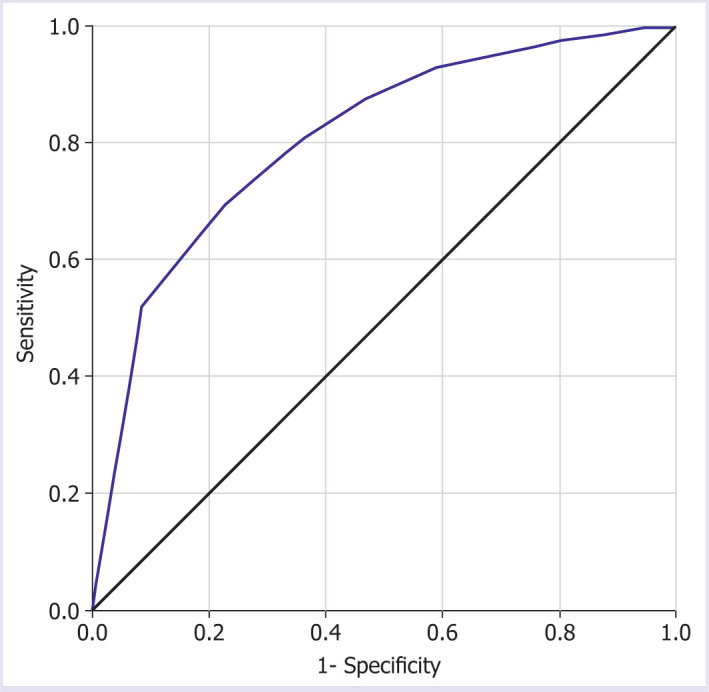
ROC analysis curve of the relationship between Charlson comorbidity index score and prognosis in COVID-19 patients.

When the possibility of CCI score predicting mortality was evaluated by ROC analysis, the area under the curve (AUC) was determined as 0.805 (95% CI: 0.748–0.861) (Fig. 2). The probability of mortality was found to be 87.2%, specificity 61.4%, positive predictive value (PPV) 18.7%, and negative predictive value (NPV) 97.9%, with a CCI score of 4 and above. If the CCI score was 5 or higher, the sensitivity was 91.5%, the specificity was 51.4%, the PPV was 22.1%, and the NPV was 97.6%.

## Discussion

It is known that patients with comorbidities are more affected by COVID-19 and are associated with worse clinical outcomes. Therefore, it is crucial to have a comprehensive assessment of comorbidities to establish the risk stratification of patients with COVID-19 on admission to the hospital [4].

During the pandemic, the severity and mortality of COVID-19 were generally related to age, gender, and the presence of comorbidities such as DM, cardiovascular, cerebrovascular, and respiratory diseases [5–7]. Advanced age and multiple comorbidities have been reported as independent mortality risk factors for COVID-19 patients [8]. According to all these studies, the CCI score was a validated, simple, and easily applicable method of estimating the risk of death from comorbid disease and is a predictor of long-term prognosis and survival.

In a meta-analysis by Kuswardhan et al. [9], it was reported that high CCI score was associated with increased mortality and disease severity in COVID-19 patients, and the risk of death increases by 16% for each increase in the CCI score. In our study, we found that there was a positive correlation (r=0.249; p<0.001) between CCI scores and mortality in COVID-19 patients, and the risk of death increased by 2.5% for each point increase in the CCI score. In our study, it was seen that the CCI score was a useful scoring system in predicting mortality. In the ROC analysis of the relationship between CCI score and mortality, AUC was found to be 0.805. CCI score of 4 and above predicted mortality with 87.2% sensitivity and 97.9% NPV.

Various biomarkers, such as CRP, D-dimer, procalcitonin, and ferritin, were frequently elevated in severe cases of COVID-19, and evaluation of these parameters might be useful in predicting serious outcomes and complications during this type of pandemic [10]. In our study, there was a positive correlation between CCI score and WBC, CRP, LDH, D-dimer, ferritin, troponin, and a negative correlation between lymphocyte count and ALT.

### Conclusion

CCI is a simple, easily applicable, and valid method for classifying comorbidities and estimating COVID-19 mortality. The close relationship between CCI score and mortality reveals the reality of how important vaccination is in patients with comorbidities.

Each point increase in CCI score increases the risk of death by 2.5%, and a CCI score of 4 and above can predict mortality with 87.2% sensitivity and 97.9% NPV. Increased awareness of potential comorbidities in COVID-19 patients can provide insight into the disease and improve outcomes by identifying and treating patients earlier and more effectively.
